# Jet-Like Appearance in Angiography as a Predictive Image Marker for the Occlusion of Intracranial Atherosclerotic Stenosis

**DOI:** 10.3389/fneur.2020.575567

**Published:** 2020-10-30

**Authors:** Xing Jin, Feina Shi, Yigang Chen, Xu Zheng, Jinhua Zhang

**Affiliations:** Department of Neurology, Sir Run Run Shaw Hospital, School of Medicine, Zhejiang University, Hangzhou, China

**Keywords:** acute ischemic stroke, large vessel occlusion, endovascular therapy, intracranial atherosclerotic stenosis, image marker, diagnostic accuracy

## Abstract

**Introduction:** Identifying intracranial atherosclerotic stenosis-related occlusion (ICAS-O) in acute ischemic stroke has important clinical significance. Correct identification would help operators devise an optimal recanalization strategy. However, it is often hard to make accurate judgments in emergency situations before thrombectomy. Here, we propose a new image marker for ICAS-O based on the appearance of occluded vessels on baseline digital subtraction angiography.

**Materials and Methods:** We retrospectively reviewed patients with acute ischemic stroke who underwent endovascular therapy from August 2017 to February 2020 at our center. ICAS-O was identified by residual focal stenosis at occluded vessels after successful recanalization. The jet-like appearance was defined as appearance of pencil-tip-like or line-linked contrast filling of the occlusion edge. A non-jet-like appearance was defined as appearance of convex, concave, or flat edge contrast filling. The proportion of jet-like appearance in different occlusion etiologies and occluded vessels was determined. The diagnostic value of jet-like appearance for ICAS-O was assessed.

**Results:** A total of 164 patients diagnosed with ICAS-O were enrolled. Jet-like appearance was detected in 34 (20.7%) patients with younger age (68.0 ± 11.9 years vs. 62.7 ± 10.2, *p* = 0.019), patients with lower baseline NIHSS scores (16.6 ± 7.1 vs. 12.4 ± 6.5, *p* = 0.002) and patients with more past stroke or transit ischemic events (31.4 vs. 13.2%, *p* = 0.011). ICAS-O rate was higher in the jet-like appearance group (82.9 vs. 8.5%, *p* < 0.001), and rescue methods were more frequently used (74.3 vs. 12.4%, *p* < 0.001). Jet-like appearance was mostly found at the origin of the middle cerebral artery (MCA) (44.1%), followed by the first segment trunk of MCA (20.6%) and internal carotid artery (ICA) supraclinoid (11.8%). Logistic regression showed that jet-like appearance was independently associated with ICAS-O [OR 180.813, 95% CI (17.966, 1,819.733), *p* < 0.001]. The sensitivity, specificity, and accuracy values for predicting ICAS-O was 96, 78, and 83%.

**Conclusion:** The jet-like appearance on the angiogram was an image marker for ICAS-O, with relatively high sensitivity and specificity, which could help operators predict underlying intracranial atherosclerotic stenosis in a timely manner and choose the optimal intervention strategy during endovascular therapy.

## Introduction

Endovascular revascularization therapy has become the first line treatment for acute ischemic stroke (AIS) with large vessel occlusion (LVO). With modern endovascular techniques, patients can achieve over 80% successful revascularization (1–7). However, good clinical outcomes represent ~50%. Clinical outcome is affected by many factors, including time from onset to recanalization, ischemic core volume, collateral compensation status, and post-procedural management. Rapid recanalization is the critical factor for achieving a favorable outcome. In this regard, one of the most important modifiable factors is to set up an optimal endovascular strategy.

Embolic occlusion (Emb-O) and intracranial atherosclerotic stenosis occlusion (ICAS-O) are two main causes of AIS with LVO, and both of them are treated with endovascular therapy in acute settings. Unlike the case with Westerners, ICAS-O is a more common cause of AIS in Asians (8). The Chinese IntraCranial AtheroSclerosis study group determined that the prevalence of ICAS defined as a ≥50% reduction in diameter on magnetic resonance angiography was 46.6% among hospitalized AIS patients (9). Another study demonstrated that at least 25% of cases of Chinese patients with anterior circulation AIS with LVO who received mechanical thrombectomy were diagnosed as ICAS-O (10). ICAS-O leads to more cases of thrombectomy failure (11–14). The underlying stenosis cannot be solved by stent retrieval; however, the irritated endothelium after thrombectomy would result in instant spontaneous re-occlusion. Accordingly, more than one third of the cases of ICAS-O ended up with rescue technique during endovascular procedure (14), including intraarterial, or intravenous GP IIb/IIIa inhibitor infusion (11, 15), balloon angioplasty, stent retriever detachment, or another stent implantation (13, 16, 17). Thus, it is very important to differentiate ICAS-O from Emb-O before starting the procedure.

A variety of methods can be used to differentiate ICAS-O from Emb-O. Clinical history includes progressive or fluctuating symptoms, low median baseline National Institutes of Health Stroke Scale (NIHSS) score, male, hypercholesterolemia, smoking; posterior circulation involvement may possibly support ICAS-O (16, 18, 19), while atrial fibrillation strongly suggests Emb-O. Imaging features include hyper-density vessel sign on non-contrast CT scan (NCCT) (20) and susceptibility vessel sign on susceptibility-weighted magnetic resonance imaging, which are specific indications of Emb-O (21). However, the identification of ICAS-O before thrombectomy is still challenging, especially in patients with complicated clinical situations. Hereby, we propose a new method simply based on the angiographic appearance of occlusion artery with high specificity for identification of ICAS-O.

## Materials and Methods

### Design and Population

We retrospectively reviewed cases of patients with AIS who underwent endovascular therapy (EVT) from August 2017 to February 2020 at our center. Patients were initially assigned to NCCT and CT angiography (<6 h) or CT perfusion (from 6 to 24 h) before EVT, based on the time from the onset of symptoms. The criteria for patients to receive EVT at our center were (1) age ≥18 years; (2) baseline NIHSS score ≥6; (3) large intracranial artery occlusion including distal internal carotid artery (ICA), first and second segment of middle cerebral artery (MCA), first segment of anterior cerebral artery (ACA), fourth segment of vertebral artery (VA), and basilar artery (BA); (4) baseline Alberta Stroke Program Early CT score (ASPECTS) ≥6, for symptoms onset within 6 h; demonstration of potentially salvageable brain tissue on CT perfusion (mismatch ratio of ≥ 1.2, absolute mismatch volume of >10 mL), as well as ischemic core <70 mL for symptoms onset more than 6 h.

First, an angiography of the target artery was used to evaluate the occluded appearance. We excluded patients without an angiography or if a clear angiography was not available prior to the thrombectomy, and patients with tandem disease or extracranial artery occlusion, which prevents visualization of the intracranial occlusion. Patients with failure of recanalization were excluded as well, since the etiology of the occlusion could not be identified. This study was approved by an institutional review committee and all participants provided their informed consent.

### Protocol for Cerebral Digital Subtraction Angiography

Simplified digital subtraction angiography (Philips FD-20, Amsterdam, Netherland or Artis zee III biplane, Siemens, Erlangen, Germany) was performed before EVT. Patients had an aortic arch PA angiogram followed by target artery PA and lateral angiograms because LVO was already identified by CT angiography (CTA) or CT perfusion (CTP). An aortic angiogram was performed with 30 ml contrast administered at 20 ml/s under 600 psi. A target artery angiogram was performed with 8 ml contrast administered at 4 ml/s for ICA and 6 ml contrast administered at 3 ml/s for vertebral artery under 300 psi. DSA images were acquired at 6 frames per second using neurointervention software.

### Image Analysis and Classification of the Etiology

**Figure 1:**
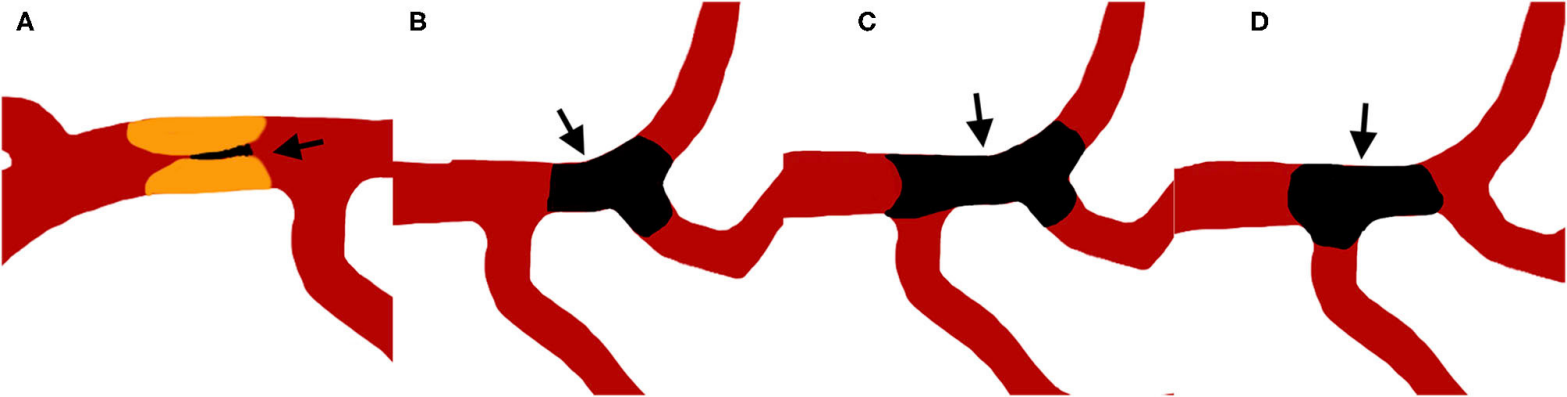
Illustration of jet-like appearance and non-jet-like appearance, arrows point to the thrombus. **(A)** shows a typical jet-like appearance of intracranial artery stenotic occlusion. **(B–D)** shows non-jet-like appearance of embolic occlusion with flat **(B)**, convex **(C)**, and concave **(D)** appearance at the proximal edge of the occluded vessel.

**Figure 2:**
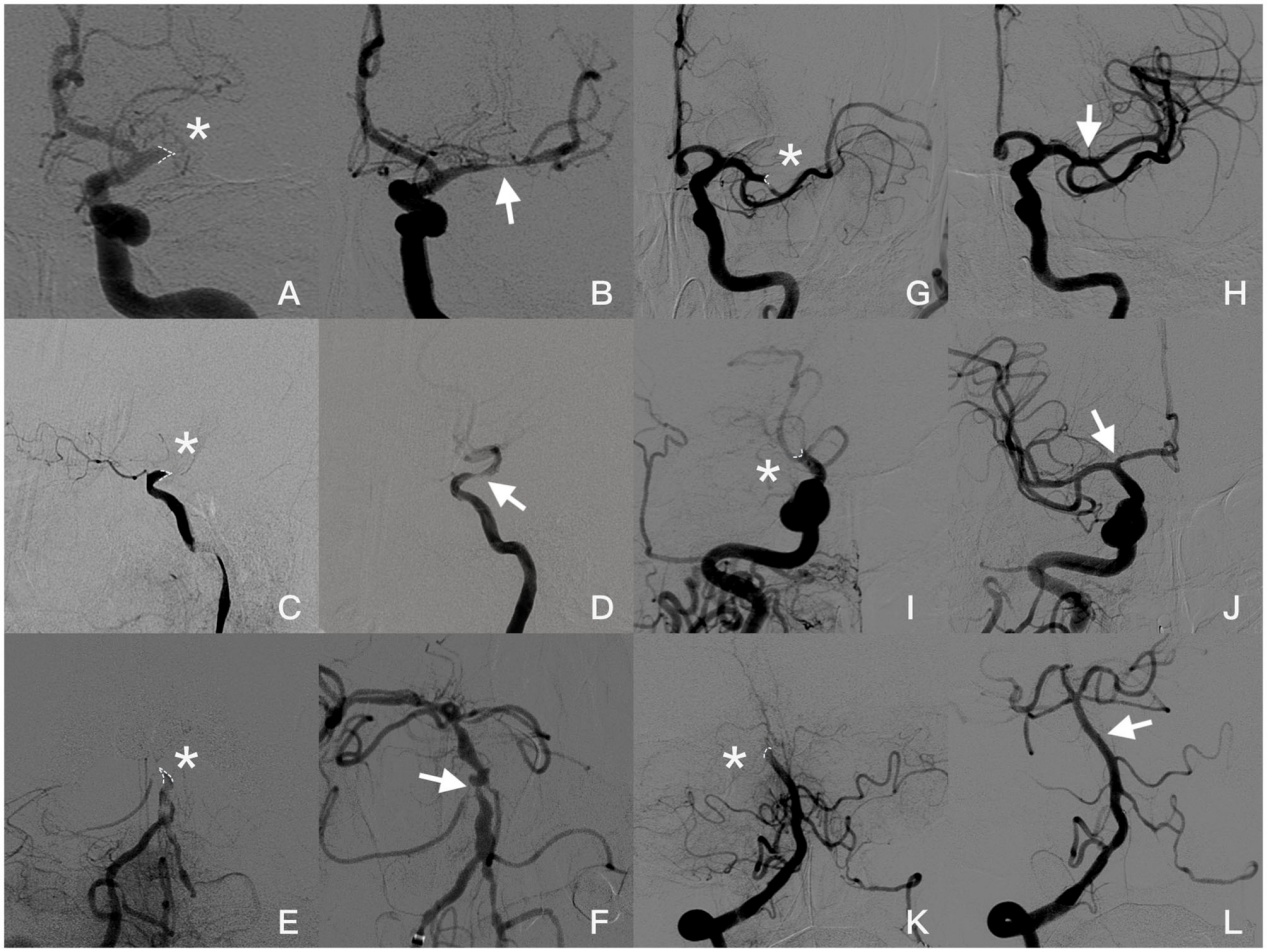
Angiogram images of a patient with jet-like appearance **(A–F)**: **(A,B)** Jet-like appearance at the origin of the left MCA in late arterial phase (asterisk). Severe stenosis (arrow) was seen in the MCA after first attempted thrombectomy. **(C,D)** Occlusion at supraclinoid of ICA presented as a jet-like appearance (asterisk). After the small-volume thrombus was removed, severe stenosis is shown (arrow) after the ophthalmic artery was taken off and forward blood flow was significantly affected. **(E,F)** Basilar artery occluded at AICA take-off (asterisk). Severe stenosis was seen after two thrombectomy attempts; however, blood flow speed was normal, and no further angioplasty was required. Angiogram images of patients without jet-like appearance **(G–L)**. **(G,H)**: Concave appearance of the occluded left MCA (asterisk), and post-thrombectomy angiography showed recanalization of the superior branch of MCA without focal stenosis. **(I,J)** Occlusion at the end of ICA with concave appearance (asterisk). Both MCA and ACA were recanalized without focal stenosis (arrow). **(K,L)** Appearance of top of basilar artery occluded (asterisk) and recanalized (arrow) in a female with atrial fibrillation. Occlusion appearance in angiography was figured out by dotted line.

Jet-like appearance was defined as the appearance of pencil-tip-like or line-linked contrast filling on the occlusion edge on DSA imaging (Figures 1A, 2A–F). The tapering segment was either near the vessel wall or in the middle of the vessel lumen. A non-jet-like appearance was defined as visualization of convex, concave, or flat edge contrast filling (Figures 1B–D, 2G–L).

Two neuroradiologists with at least 10 years of experience, blinded to the clinical information, retrogradely reviewed the initial angiograms and classified the appearance of the occlusions. Another neuroradiologist would determine the classification if a consensus could not be reached.

Occlusion etiology was classified as intracranial atherosclerotic stenosis occlusion (ICAS-O) or Emb-O. ICAS-O was identified by residual fixed stenosis >70% or lower-degree stenosis with a tendency for re-occlusion or flow impairment during the procedure (13). Emb-O was identified by a lack of fixed focal stenosis after successful recanalization, or temporary stenosis that recovered in angiography 20 min later without angioplasty (13).

### Statistics

The analysis was performed using the SPSS 24.0 statistical program (IBM SPSS, Armonk, NY). Continuous values are expressed as median and inter-quartile range (IQR), and categorical values are expressed as counts and percentage. Continuous variables were compared with the Mann–Whitney test, and categorical variables were compared using the Chi square test or Fisher's exact test. Statistical significance was defined as *p* < 0.05 and variables with *p* < 0.1 on univariate analysis were included in logistic regression. Diagnostic parameters, including sensitivity, specificity, and accuracy value, were calculated to assess the value of the jet-like appearance when differentiating the etiology. The kappa statistic value was used to assess intraobserver and interobserver variability when assessing the jet-like appearance.

## Results

A total of 249 patients with LVO underwent thrombectomy during this period. After reviewing the images, 66 patients were excluded (49 patients did not have clear angiogram images, 13 patients had tandem disease, and 4 patients suffered severe intracranial hemorrhage and thrombectomy were ceased). A total of 183 patients were finally analyzed, 27 (14.7%) patients were under local anesthesia due to severe cardiac or pulmonary disease, 164 patients (93 males, mean age 66.8 ± 11.8 years, mean initial NIHSS 15.7 ± 7.2) achieved successful recanalization (final mTICI ≥ 2b). Among them, 42 (25.6%) suffered internal carotid artery occlusion, 98 (59.8%) had MCA occlusion, 18 (11.0%) had vertebral BAs occlusion, 40 (24.4%) were identified as ICAS-O, and 124 (75.6%) were identified as Emb-O.

Example illustrations of patients with and without jet-like appearance were shown in Figure 1. Jet-like appearance was observed in 34 (20.7%) patients. The intraobserver and interobserver kappa values for detection of jet-like appearance were 0.889 and 0.847, respectively.

As Table 1 shows, patients with jet-like appearance were younger (68.0 ± 11.9 years vs. 62.7 ± 10.2, *p* = 0.019), and had lower baseline NIHSS scores (16.6 ± 7.1 vs. 12.4 ± 6.5, *p* = 0.002). A history of past stroke and transient ischemic attack was more frequent in patients with jet-like appearance (*p* = 0.011), and atrial fibrillation was more common in patients without jet-like appearance (*p* = 0.001). Stroke etiology was distributed differently in the two groups. Cardiac embolism was the most common etiology for patients without jet-like appearance. For patients with jet-like appearance, 71.4% were classified as having large artery atherosclerosis. The ICAS-O rate was higher in the jet-like appearance group as well (82.9 vs. 8.5%, *p* < 0.001), and rescue methods were more frequently used, including intraarterial tirofiban infusion, balloon angioplasty, and stent placement (*p* < 0.001).

**Table 1:** Comparison of baseline characteristics between patients with and without Jet-like appearance.

**Variables**	**No jet-like appearance (*n* = 129)**	**Jet-like appearance (*n* = 35)**	***p*-value**
Age, year	68.0 ± 11.9	62.7 ± 10.2	0.019
Male, *n* (%)	69 (53.4%)	24 (68.6%)	0.110
Baseline NIHSS score	16.6 ± 7.1	12.4 ± 6.5	0.002
ASPECTS, median (IQR)	8 (6–9)	8 (7–9)	0.124
**Past medical history**	17 (13.2)	11 (31.4)	0.011
Stroke/TIA, *n* (%)			
Atrial fibrillation, *n* (%)	75 (58.1)	9 (25.7)	0.001
Hypertension, *n* (%)	85 (65.9)	25 (71.4)	0.536
Diabetes, *n* (%)	22 (17.1)	8 (22.9)	0.431
Hyperlipidemia, *n* (%)	26 (20.2)	6 (17.1)	0.690
TOAST etiologies			<0.001
LAA, *n* (%)	10 (7.8)	25 (71.4)	
CE, *n* (%)	90 (69.8)	5 (14.3)	
Other, *n* (%)	4 (3.1)	0 (0)	
UE, *n* (%)	25 (19.4)	5 (14.3)	
Occlusion type			<0.001
ICAS-O, *n* (%)	11 (8.5)	29 (82.9)	
Emb-O, *n* (%)	118 (91.4)	6 (17.1)	
Door-Puncture time (min)	147.5 ± 61.4	153.8 ± 60.4	0.605
Puncture-Recanalization time (min)	74.9 ± 53.0	84.2 ± 65.7	0.424
Rescue methods			<0.001
None, *n* (%)	113 (87.6)	9 (25.7)	
IA Tirofiban, *n* (%)	4 (3.1)	3 (8.6)	
Balloon, *n* (%)	3 (2.3)	8 (22.9)	
Stent, *n* (%)	9 (7.0)	15 (42.9)	
SIH, *n* (%)	9 (7.0)	1 (2.9)	0.366
90d mRS, median (IQR)	3 (1–5)	2 (1–5)	0.414

*NIHSS, National Institutes of Health Stroke Scale; LAA, large artery atherosclerosis; CE, cardiac embolism; UE, undetermined etiology; ICAS-O, intracranial atherosclerotic stenosis occlusion; Emb-O, embolic occlusion; IA, intraarterial; SIH, symptomatic intracranial hemorrhage; mRS, Modified Rankin Scale. Data was expressed as mean ± standard deviation for continuous variables*.

The proportion of jet-like appearance and ICAS-O differed according to the occlusion site (Table 2). Jet-like appearance was mostly found at the origin of the MCA (44.1%), followed by the first segment trunk of the MCA (20.6%) and the supraclinoid of the ICA (11.8%). There was no jet-like appearance in the petro-cavernous segment of the ICA. In comparison, the origin of the MCA (35.0%), first segment trunk of the MCA (25.0%) and supraclinoid of the ICA (10.0%) were the most common sites for ICAS-O.

**Table 2:** Proportion of jet-like appearance and ICAS-O at different occlusion sites.

**Occlusion site**	**Jet-like appearance (*n* = 34)**	**ICAS-O** **(*n* = 40)**	**Emb-O** **(*n* = 124)**
ICA, *n* (%)			
Supraclinoid	4 (11.8)	4 (10.0)	19 (15.3)
Petro-cavernous	0 (0)	2 (5.0)	15 (12.1)
MCA, *n* (%)			
Origin	15 (44.1)	14 (35.0)	8 (6.5)
M1 trunk	7 (20.6)	10 (25.0)	26 (21.0)
M1 branch	3 (8.8)	3 (7.5)	26 (21.0)
VBA, *n* (%)			
VA after PICA	2 (5.9)	2 (5.0)	0 (0)
BA origin	1 (2.9)	1 (2.5)	1 (0.8)
BA after AICA	2 (5.9)	2 (5.0)	11 (8.9)
Other vessels, *n* (%)	0 (0)	0 (0)	18 (15.2)

*ICAS-O, intracranial atherosclerotic stenosis occlusion; Emb-O, embolic occlusion; ICA, internal carotid artery; MCA, middle cerebral artery; M1, first segment of middle cerebral artery; VBA, vertebral basilar artery; VA, vertebral artery; PICA, posterior inferior cerebellar artery; BA, basilar artery; AICA, anterior inferior cerebellar artery*.

Thus, age, baseline NIHSS score, atrial fibrillation, history of stroke/TIA, and jet-like appearance were included in a binary logistic regression model to select predictors for ICAS-O. Binary logistic regression analysis (Table 3) revealed that jet-like infusion sign was independently associated with ICAS-O, after adjusting for age, baseline NIHSS score, atrial fibrillation, and history of stroke/TIA [OR 180.813, 95% CI (17.966, 1,819.733), *p* < 0.001].

**Table 3:** Univariant and multivariant logistic regression of predictors for ICAS-O.

**Variables**	**Unadjusted OR (95% CI)**	**Adjusted OR (95% CI)**
Age	0.946 (0.916, 0.977)	1.019 (0.957, 1.084)
Baseline NIHSS score	0.846 (0.785, 0.912)	0.826 (0.718, 0.949)
Atrial fibrillation	0.061 (0.020, 0.183)	0.011 (0.001, 0.142)
History of stroke/TIA	2.893 (1.229, 6.810)	4.638 (0.620, 34.686)
Jet-like appearance	51.848 (17.705, 151.835)	180.813 (17.966, 1,819.733)

*ICAS-O, intracranial atherosclerotic stenosis occlusion; NIHSS, National Institutes of Health Stroke Scale; TIA, transient ischemic attack; OR, odds ratio; CI, confidence interval*.

Diagnostic indices are shown in Table 4. The sensitivity, specificity, and accuracy values for the jet-like infusion sign for predicting ICAS-O were 73, 95, and 90%, respectively, for all patients with ICAS-O. The accuracy of predicting ICAS-O at the origin of the MCA, and supraclinoid of the M1 trunk and ICA was 96, 78, and 83%, respectively.

**Table 4:** Diagnostic testing of Jet-like appearance for predicting ICAS-O.

	**Jet-like infusion sign, n**	**Sensitivity 95% CI**	**Specificity 95% CI**	**Positive predictive value, 95% CI**	**Negative predictive value, 95% CI**	**Accuracy**
All patients (*n* = 164)	34	0.73 (0.56, 0.85)	0.95 (0.90, 0.98)	0.83 (0.68, 0.92)	0.92 (0.87, 0.95)	0.90 (0.84, 0.94)
MCA origin occlusion (*n* = 22)	15	1.00 (0.77, 1.00)	0.87 (0.47, 1.00)	0.93 (0.69, 0.99)	1.00	0.96 (0.77, 1.00)
M1 trunk (*n* = 36)	7	0.40 (0.12, 0.74)	0.88 (0.70, 0.98)	0.57 (0.26, 0.83)	0.79 (0.69, 0.87)	0.75 (0.58, 0.88)
ICA supraclinoid (*n* = 23)	4	0.50 (0.07, 0.93)	0.89 (0.67, 0.98)	0.17 (0.05, 0.39)	0.50 (0.16, 0.84)	0.83 (0.61, 0.95)

*ICAS-O, intracranial atherosclerotic stenosis occlusion; MCA, middle cerebral artery; M1, first segment of middle cerebral artery; ICA, internal carotid artery*.

## Discussion

Here, we proposed that the jet-like appearance at an occluded artery on angiography is an independent image marker for ICAS-O. Out of all patients with LVO, jet-like appearance was detected in 73% of patients with ICAS-O, and exhibited 95% specificity for ICAS-O, suggesting that the jet-like appearance is useful for predicting ICAS-O before thrombectomy.

In our study, 24.4% of patients were determined as having ICAS-O, which was consistent with previous studies (10, 22). Patients with ICAS-O presented the same features as other studies, like younger age (17) and lower baseline NIHSS scores (16, 19). Furthermore, a history of stroke or TIA was more common among ICAS-O patients, while atrial fibrillation was more common in Emb-O patients.

Patients with jet-like appearance and ICAS-O shared similar characteristics of their medical histories and baseline NIHSS scores. After adjusting for other confounding factors, this study demonstrated that jet-like appearance before thrombectomy strongly indicated ICAS-O. Furthermore, the predictive value of jet-like appearance differed according to the occlusion site. Jet-like appearance was easily found where the occlusion occurred distal to a large branch take-off, such as the ICA supraclinoid (where ophthalmic artery take-off was performed), origin of the MCA (where ACA take-off was performed), or the fourth segment of the vertebral artery (where posterior inferior cerebellar artery take-off was performed). For occlusions occurring proximal to a large branch take-off, contrast agent was insufficient to reach the occlusion edge. This could explain why jet-like appearance was never seen in our study at the petro-cavernous segment of the ICA, where atherosclerotic stenosis frequently occurs. It also explained the low sensitivity of jet-like appearance in the trunk of the MCA first segment.

Occlusion caused by intracranial arterial dissection (ICAD) sometimes demonstrates a jet-like appearance and would be difficult to discriminate. “Intimal flap” and “double lumen” are the most characteristic findings associated with ICAD (23). However, these findings are visible in only a few cases. The “pearl and string sign” and retention of contrast media in the false lumen are often seen in conventional cerebral angiographies of ICAD. Furthermore, whether or not a fixed focal stenosis by single-stent retriever deployment in the occlusion can be the criterion for differentiating ICAD from ICAS-O.

Stent retrievers and contact aspiration devices both demonstrate significant efficacy as first line methods for mechanical thrombectomy (24). However, both are primarily designed for embolism occlusion rather than ICAS-O (7, 25, 26). Stent retriever thrombectomy often results in instant spontaneous re-occlusion due to subsequent thrombosis at the site of ICAS (11). On the other hand, contact aspiration seems less effective than stent retrievers for ICAS-O, resulting in a longer time from puncture to reperfusion, longer procedure duration, and a higher rate of switching to an alternative thrombectomy technique (27, 28). As mentioned above, ICAS-O often requires intraarterial or intravenous GP IIb/IIIa inhibitor infusion, emergent balloon angioplasty, and stenting (11, 15–17, 29). Intraarterial infusion of GP IIb/IIIa inhibitor tirofiban at the occlusion site may be a reasonable therapeutic option since the major components of thrombi *in situ* of ICAS are rich in platelets and fibrin. Recent studies have shown that both intracranial angioplasty/stenting and intraarterial infusion of a glycoprotein IIb/IIIa inhibitor are effective and safe in the treatment of AIS patients with ICAS-O (30). In ACTUAL study, investigators found that patients with acute anterior ICAS-O, who received primary angioplasty treatment, showed favorable independent outcomes at 90 days and lower rates of asymptomatic intracranial hemorrhage compared to patients who received primary stent retriever thrombectomy (31). All this evidence suggest that the optimal early endovascular strategy for ICAS-O is quite different than that for Emb-O. Thus, early identification of ICAS-O may help operators modify the treatment approach before initiating intervention. Operators should reduce thrombectomy passes and switch to rescue methods in a timely manner once ICAS-O is determined. However, it is difficult for operators to make accurate judgment in emergent situations due to incomplete clinical information. Coexistence of ICAS and atrial fibrillation further increases the difficulty. Deng et al. (10) determined that 8.6% of anterior circulation LVO patients who received mechanical thrombectomy had both of them. In our study, the corresponding ratio was 1.8% (3/164). Yet, all 3 patients showed jet-like appearance and were classified as ICAS-O, indicating that jet-like appearance can predict ICAS-O effectively, even in complex clinical situations.

Occlusion type is a highly specific image marker for differentiating ICAS-O from Emb-O. Truncal type occlusion during thrombectomy post-stent deployment had 87.4% specificity to predict fixed focal stenosis (32). Microcatheter first-pass effect, a string-like blood flow observed in angiography after the microcatheter is retrieved from the occlusive vessel segment, indicates ICAS-O (33). First-pass effect can accurately predict ICAS-O in 88.5% of patients with LVO (33). These methods have a few limitations despite their high specificity. The main problem is that additional manipulations are required to determine the occlusion etiology; for example, microcatheter angiography beyond the occlusion, post-deployment angiography, or repeated microcatheter movement in the occlusion. All these manipulations implied the risk of irritating the inflamed plaque in ICAS-O (34). Besides, occlusion type practically depends on stent-through blood flow and cannot be determined in patients undergoing non-stent thrombectomy. In comparison, the jet-like appearance can help interventionists identify ICAS-O before the procedure, so that they can choose the most appropriate frontline instruments and strategies. Occlusion type based on CTA was another applicable method for determining ICAS-O before thrombectomy. However, conventional CTA imaging may vary depending on whether the scan phase is arterial, arteriovenous, or venous-weighted (35, 36). Unequal CTA quality would make ICAS-O identification difficult. Multiphase CTA or CTP could resolve this shortcoming but is not available at most centers (3, 37–40).

Our study has several limitations. Firstly, this study was performed at a single stroke center, with a relatively small number of ICAS-O patients. Secondly, 49 patients were excluded because of a lack of clear angiogram images. This accounts for 26.5% and may limit the level of rigor of the data. Among them, 35 patients did not undergo a first angiography, but a roadmap was available. A roadmap often shows images of the vessels in early arterial phase for clear navigation, while occlusion edges on DSA imaging can only be observed clearly in the late arterial phase due to decreased blood flow motivation. Furthermore, a roadmap is often not available for patients undergoing local anesthesia. Therefore, in our opinion, a clear angiography including complete arterial and venous phase would be crucial for detecting jet-like appearance. The main drawback of jet-like appearance is that it relies on the occlusion site and hemodynamic status. Jet-like appearance is rarely shown in the petro-cavernous segment of the ICA or the origin of the BAs. Therefore, jet-like appearance is not an appropriate marker for predicting ICAS-O in these areas. Finally, a larger sample size and multicenter study are required to verify the predictive value of jet-like appearance for ICAS-O.

## Conclusion

The present study proposed jet-like appearance in angiogram as an image marker for ICAS-O, with relatively high sensitivity and specificity. This could help operators predict underlying intracranial atherosclerotic stenosis in a timely manner and choose the best intervention strategy during thrombectomy.

## Data Availability Statement

The raw data supporting the conclusions of this article will be made available by the authors, without undue reservation.

## Ethics Statement

The studies involving human participants were reviewed and approved by Sir Run Run Shaw Hospital Ethics Committee School of Medicine, Zhejiang University. The patients/participants provided their written informed consent to participate in this study. Written informed consent was obtained from the individual(s) for the publication of any potentially identifiable images or data included in this article.

## Author Contributions

JZ and XJ proposed the concept and designed the study. XJ, YC, and XZ performed data collection and image analysis. FS and XJ performed statistical analysis. XJ and JZ drafted the paper, and JZ approved the final paper for publication. All authors contributed to the article and approved the submitted version.

## Conflict of Interest

The authors declare that the research was conducted in the absence of any commercial or financial relationships that could be construed as a potential conflict of interest.
